# Prevalence and Factors Associated With Tinea Pedis Among Diabetic Patients in Saudi Arabia: A Descriptive Cross-Sectional Study

**DOI:** 10.7759/cureus.51210

**Published:** 2023-12-28

**Authors:** Nouf Alhammadi, Marwah AL-Jallal, Hanan A AlKaabi, Ward M Malibari, Rahaf S Al Jallal, Abdulmalik S Almarshad, Fahad H Binshalhoub, Amirah N Albalawi, Tahani A Adam, Alaa H Al-Khairat

**Affiliations:** 1 Rheumatology, King Khalid University, Abha, SAU; 2 Medicine, King Khalid University, Abha, SAU; 3 Medicine, Umm Al-Qura University, Makkah, SAU; 4 Radiology, Applied Medical Sciences, King Khalid University, Abha, SAU; 5 Medicine, Qassim University, Buraydah, SAU; 6 Medicine and Surgery, Imam Mohammad Ibn Saud Islamic University, Riyadh, SAU; 7 Medicine, University of Tabuk, Tabuk, SAU; 8 Statistics and Operation Research, Qassim University, Buraydah, SAU; 9 Medicine, Jazan University, Jazan, SAU

**Keywords:** diabetes mellitus, saudi arabia, risk factors, prevalence, fungal infections, tinea pedis

## Abstract

Background: It has been estimated that 30% of diabetic people experience dermatological problems. Fungal infections are the most frequent cause of these lesions. While tinea infections in non-diabetic individuals rarely cause symptoms, in diabetes patients, they can create fistulas and entry sites that can result in catastrophic bacterial infections.

Aim: This research paper aims to evaluate the prevalence and factors associated with tinea pedis among diabetic patients in Saudi Arabia.

Methods: The research paper incorporated a cross-sectional study approach with the involvement of a questionnaire-based response aimed at all Saudi inhabitants with diabetes mellitus (DM) who conceded to be part of the study. This research was carried out from March 22, 2023, to May 22, 2023, spanning for three months. The participants who satisfied our requirements provided data using computerized Google Forms for data collection; no nominative information disseminated via social media platforms was visible. The three components of the questionnaire address diabetic information, tinea pedis infections, and foot care.

Results: A total of 295 people with diabetes case were involved in the study. Among them, 149 (50.5%) were males, and their ages stretched from 16 to above 60 years, with a mean age of 49.5 ± 12.9 years old. A total of 194 (65.8%) of the study patients had type II DM. Of 134 (45.4%) were diagnosed with diabetes for more than 10 years. Exact 152 (52%) of the study diabetic patients were diagnosed with tinea pedis. Only patients' BMI showed a significant association with having tinea pedis as 47 of overweight diabetics were diagnosed with tinea pedis versus 47 of obese patients and only five patients of others who were underweight (p=0.049).

Conclusion: This research concluded that almost 50% of patients with diabetes were suffering from obesity and were earlier diagnosed with tinea pedis and poor glycemic control irrespective of reported good diabetic foot care.

## Introduction

Diabetes is among the important chronic and metabolic diseases that are progressively becoming more prevalent in diabetes mellitus [1,2]. About 30% of diabetes individuals experience different skin lesions, and fungal skin infections make up a sizable portion of these lesions [3]. Chronic hyperglycemia in patients is thought to disrupt polymorphonuclear leukocytes, cellular immunity, and phagocytic activities. Patients with this syndrome frequently acquire cutaneous fungal infections, as well as other bacterial illnesses [4,5].

Just like onychomycosis, tinea pedis is an easily transmitted fungal disease that infects 15% to a fifth of the overall population [6-8]. Though the adverse effects associated with tinea pedis make an individual restless and cosmetically not connected, it is inappropriate to be linked with any critical adverse complications mainly among fully immune persons [9,10]. Individuals ailing from diabetes have higher chances of contracting tinea pedis due to factors such as compromised immune function, poor circulation, and nerve damage [11,12]. Tinea pedis increases the likelihood of bacterial infections, which can result in conditions such as cellulitis, ulcers, gangrene, osteomyelitis, and, in severe cases, potential lower limb amputation [13,14]. To effectively manage tinea pedis in diabetic patients, treatment strategies may include the use of antifungal medications, topical creams or ointments, and proper foot hygiene. However, studies focusing on the success of treatment, specifically in individuals affected by diabetes with tinea pedis, are limited; hence, further studies are needed to optimize treatment approaches [15,16].

Diabetes patients who develop onychomycosis and tinea pedis frequently experience relapse and reinfection because of monopathy, vasculopathy, and neuropathy [17]. Numerous studies compare the frequency of tinea pedis and onychomycosis in diabetic individuals to the general population and find either a similar or greater rate. The current study mainly aimed to establish the incidence of influence related to tinea pedis in individuals suffering from diabetes.

## Materials and methods

Study design and setting

This was a cross-sectional conducted among diabetic patients within the Saudi population. This research was carried out from March 22, 2023, to May 22, 2023, spanning three months.

Inclusion and exclusion criteria

We included all diabetic patients living in Saudi Arabia who consented to be part of the study. The target population was respondents aged between 16 and 60 years. We excluded people outside the age range and people who did not consent to undertake the study.

Sampling technique

We implemented the simple random sampling technique to ensure that every respondent had an equal chance of selection. The primary purpose of employing simple random sampling is to enable unbiased generalizations about Saudi diabetic patients. The sample size was determined using sample Cochran's formula, using a 95% CI, a 6% margin of error, and an assumed prevalence rate of 50%. The calculation resulted in a minimum sample size of 267 participants.

Data collection/research tool

Participants meeting the criteria for electronic data collection were engaged through specifically designed forms, ensuring no external influence from social media. To refine the questionnaire's clarity, validity, and reliability, a meticulous testing phase involved a maximum of three respondents providing feedback. Their input-shaped enhancements were incorporated into the final questionnaire, structured into three core sections. The initial segment focused on gathering demographic data, followed by a closed-format inquiry on diabetes in the second section. The third section centered on the diagnosis of tinea pedis and foot hygiene practices. Qualified respondents were encouraged to exhaust their input, contributing comprehensive information for this research.

Data analysis

After data collection, the data were cleaned using Microsoft Excel and analyzed using the Statistical Product and Service Solutions (SPSS) version 26 (IBM Corp., Armonk, NY). Frequencies and percentages were obtained using descriptive statistics. Descriptive analysis was done by computing frequencies and percentages. The occurrence of tinea pedis on infected individuals was presented in graphs. Cross tabulation for showing factors associated with having tinea pedis among the diabetics was calculated using the chi-square test and exact probability test in instances where the sample size was small.

Ethical considerations

Ethical approval for this research was given by the King Khalid University (IRB No: 2023-1107). Informed consent and voluntary willingness to participate in this study were some of the principles considered during data collection in this study. Respondents were clearly informed of the risks and benefits of their participation in this study prior to engagement. Only the researchers can access the information of patients. The publication only presented a summary of the statistics and will not be used personal information.

## Results

An overall of 295 people with diabetes case participated in this research. A total of 149 (50.5%) were males, and their ages stretched from 16 to above 60 years, with a mean age of 49.5 ± 12.9 years old. A total of 279 (94.6%) were Saudi, 98 (33.2%) from the western region, 74 (25.1%) from the southern region, and 74 (25.1%) from the eastern region. As for education, 164 (55.6%) were graduates from tertiary institutions, 53 (18%) had achieved high school standards of education, and 40 (13.6%) had post-graduate degrees. Exact 139 (47.1%) were retired, 58 (19.7%) were teachers, and 47 (15.9%) were students. A total of 105 (36.6%) were obese, and 96 (32.5%) were overweight (Table 1). 

**Table 1:** Demographic characteristics of the participants.

Demographic variable	Category	Count	Percent
Gender	Female	1657	61.2
	Male	1052	38.8
Age (years)	<20	291	10.7
	20-30	1253	46.3
	31-40	521	19.2
	41-50	310	11.4
	51-60	193	7.1
	>60	141	5.2
Nationality	Saudi	2493	92.0
	Non-Saudi	216	8.0
Place of residency	Western Region	815	30.1
	Southern Region	555	20.5
	Northern Region	120	4.4
	Middle Region	664	24.5
	Eastern Region	555	20.5
Education	Illiterate	34	1.3
	Primary	44	1.6
	Intermediate	84	3.1
	Secondary	614	22.7
	University	1780	65.7
	Postgraduate	153	5.6
Occupation	Student	1153	42.6
	Teacher	303	11.2
	Military	34	1.3
	Housewife	102	3.8
	Doctors	92	3.4
	Engineer	154	5.7
	Retired	864	31.9
	Others	7	.3
BMI	<18.5	238	8.8
	18.5-24.9	1218	45.0
	25-29.9	640	23.6
	>=30	613	22.6

Regarding diabetes data (Table 2), 194 (65.8%) of the study patients had type II DM. Exact 134 (45.4%) were diagnosed with diabetes for more than 10 years, while 73 (24.7%) had diabetes for one to five years. As for HbA1c, it was 6.6-7.5% among 104 (35.3%) and above 7.5% among 44.4%.

**Table 2:** Characteristics of diabetic patients.

Clinical characteristics	Category	Count	Percent
Type of diabetes	Type 1	101	34.2
	Type 2	194	65.8
Duration of DM	<1 year	31	10.5
	1-5 years	73	24.7
	6-10 years	57	19.3
	>10 years	134	45.4
Average of your HbA1c	<=6.5%	60	20.3
	6.6-7.5%	104	35.3
	7.6-8.5%	69	23.4
	8.6-9.5%	38	12.9
	>=9.6%	24	8.1

As for foot hygiene practices for diabetic patients (Table 3), more than half of the study patients (55.6%) examine their feet regularly, 89.2% use nail scissors regularly, and 52.9% wear socks regularly. A good number of respondents (70.2%) attested that they washed their feet three times per day.

**Table 3:** Foot hygiene practices for diabetic patients.

Practice		Count	Percent
Examine feet regularly		164	55.6
Use nail scissors regularly		263	89.2
Wearing socks regularly		156	52.9
Wearing socks	Frequently	0	0
	During winter	192	65.1
	Rarely	103	34.9
How many times do you wash your feet daily?	Three times or more	207	70.2
	Less than three times	88	29.8

**Figure 1:**
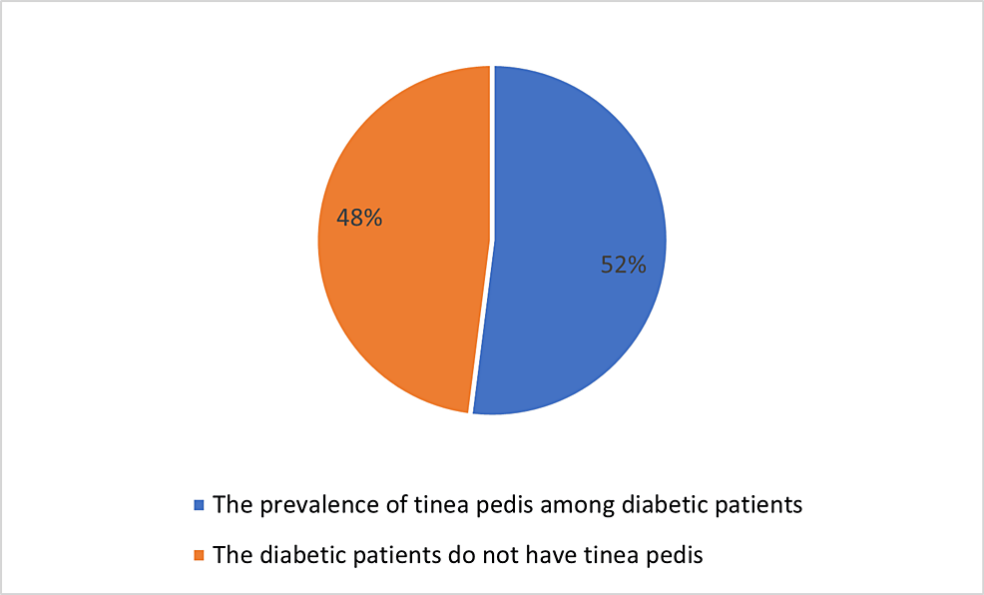
The prevalence of diabetes among patients with tinea pedis and patients without tinea pedis.

With regard to the prevalence of tinea pedis among diabetic patients (Figure 1), it was clear that 152 (52%) of the study diabetic patients were diagnosed with tinea pedis.

Only patients' BMI showed a significant association with having tinea pedis as 47 of overweight diabetics were diagnosed with tinea pedis versus 47 of obese patients and only five patients of others with underweight (p=0.049). All other demographic factors showed insignificant association with experiencing tinea pedis among the study patients (Table 4).

**Table 4:** Comparison of different demographic variables using chi-square testing. Social demographic characteristics are presented in frequencies (n) and proportion (%). The p-value is considered statistically significant at p<0.05. BMI - Body Mass Index

Social demographic data	Category	Were you diagnosed with tenia pedis?		P-value
		Yes	No	
	Female	77	49	0.441
	Male	75	58	
Age (years)	<20	7	6	0.992
	20-30	24	15	
	31-40	22	14	
	41-50	23	16	
	51-60	40	28	
	>60	36	28	
Nationality	Saudi	146	100	0.346
	Non-Saudi	6	7	
Place of residency	Western Region	57	35	0.363
	Southern Region	27	29	
	Northern Region	7	3	
	Middle Region	15	13	
	Eastern Region	46	27	
Education	Illiterate	7	6	0.892
	Primary	3	3	
	Intermediate	8	4	
	Secondary	26	23	
	University	88	56	
	Postgraduate	20	15	
Occupation	Student	27	13	0.496
	Teacher	32	16	
	Military	1	3	
	Housewife	5	3	
	Doctors	3	3	
	Engineer	16	13	
	Retired	67	56	
	Others	1	0	
BMI	<18.5	5	7	0.063
	18.5-24.9	53	22	
	25-29.9	47	42	
	>=30	47	36	

As for the reaction between having tinea pedis and diabetes (Table 5), only patients with HbA1c showed a significant association where 24 of diabetic patients with HbA1c had tinea pedis of 7.6-8.5% compared to 59 of others with HbA1c of 6.6-7.5% and 39 of others with HbA1c less than 6.5% (p=0.038). Table 6 shows the association between tinea pedis among diabetic patients and their foot hygiene practice. Exact 131 of those who use ail scissors regularly had tinea pedis compared to 21 others who did not (p=0.047).

**Table 5:** Comparison of different characteristics of diabetic patients using chi-square testing. Characteristics of diabetic patients are presented in frequencies (n) and proportion (%). The p-value is considered statistically significant at p<0.05. DM - Diabetes mellitus, HbA1c - Hemoglobin A1C (blood test).

Characteristics of diabetic patients	Category	Were you diagnosed with tenia pedis?		P-value
		Yes	No	
Type of diabetes	Type 1	53	34	0.604
	Type 2	99	73	
Duration of DM	<1 year	22	6	0.153
	1-5 years	37	27	
	6-10 years	28	21	
	>10 years	65	53	
Average of your HbA1c	<=6.5%	39	20	0.038
	6.6-7.5%	59	30	
	7.6-8.5%	24	33	
	8.6-9.5%	18	14	
	>=9.6%	12	10	

## Discussion

Tinea pedis is a frequent fungal infection mainly among diabetic patients who have a higher likelihood due to their underlying health conditions. It is important for diabetic patients to be proactive in preventing and treating tinea pedis to avoid complications and maintain foot health [18-20].

The current research was purposing to evaluate the occurrence of tinea pedis in individuals infected with diabetes in Saudi Arabia and its associated factors. The study indicated that more than 50% of the individuals ailing from diabetes were also infected with tinea pedis. Higher prevalence was significantly associated with overweight/obesity, poor foot hygiene practice, and uncontrolled blood glucose levels. This high prevalence may be due to the dominance of type 2 diabetes mellites with long-disease duration, which exceeded 10 years in most cases. Additionally, most cases showed high HbA1c levels, indicating poor diabetic control. The surprising finding was the high prevalence of tinea pedis irrespective of a reported satisfactory level of foot care, which may be biased by subjective reports by patients who may be embarrassed to tell the truth about their actual foot care practice [20].

Studies have shown that the prevalence of tinea pedis among diabetic patients can be significant. In one study, the prevalence of onychomycosis (fungal infection of the nails) and tinea pedis in diabetic patients was found to be 40.6% and 10.9%, respectively [21]. Another study found that tinea pedis and onychomycosis occurred in approximately one-third of diabetic patients with foot ulcers [22]. A non-controlled study revealed that the prevalence of onychomycosis among diabetic patients was 22% [23].

Svejgaard et al. revealed that the prevalence of onychomycosis among people of a similar age in Western Europe was 4.9% and that diabetes is a metabolic illness that makes onychomycosis more likely [24]. In Saudi Arabia, Alqahtani et al. documented that 11% of type 2 diabetic patients had tinea pedis [25]. About one-third of the respondents were aged 60 years and above; most of them used nail scissors regularly and washed their feet more than three times daily, and 71.5% of the patients examined their feet and toes. Additionally, 82.3% did not have tinea pedis before, and 48.5% had a doctor examine their feet. In the Al-Qassim region, Shahzad et al. found that exactly 80.6% whose diabetes was less than five years had skin manifestations, including fungal infections, compared to 98% of others having had diabetes for more than five years [26].

Toukabri et al. researched the prevalence and risk factors of tinea pedis in diabetes patients in Tunisia [27]. According to their study, 98.1% of the patients had Trichophyton rubrum, and 70.5% of the patients had dermatophytes. The study further found that ritual washing and communal showers are predictors of tinea pedis. The biology and etiology of skin problems in persons with diabetes were addressed by de Macedo et al. [28]. Additionally, it was demonstrated that individuals with diabetes typically experience skin lesions, with diabetic foot ulcers being mostly caused by uncontrolled blood glucose levels. The skin complications may be caused by various factors such as impaired circulation, damage of nerves, and compromised immune function. Adequate skin hydration and prompt treatment of any skin conditions are the most crucial elements in the management of skin diseases in diabetes patients.

The significant constraint and limitation in this investigation was the employment of a cross-sectional study design that can only establish the relations between factors but not causalities. As this was a survey-based study, recollection bias might be a limitation that also needs further investigation.

## Conclusions

This research concluded that almost 50% of patients with diabetes were suffering from obesity and were earlier diagnosed with tinea pedis and poor glycemic control, irrespective of reported good diabetic foot care. Consequently, the authors recommended that the incidence and pattern of fungal infections and tinea pedis, as an important cause of morbidity in diabetes, should regularly be surveyed, any infections should be properly managed, and patients' awareness regarding the protective measures and risk factors should be enhanced.
